# Role of Hydrogen Sulfide in the Pathology of Inflammation

**DOI:** 10.6064/2012/159680

**Published:** 2012-10-09

**Authors:** Madhav Bhatia

**Affiliations:** Department of Pathology, University of Otago, P.O. Box 4345, Christchurch 8140, New Zealand

## Abstract

Hydrogen sulfide (H_2_S) is a well-known toxic gas that is synthesized in the human body from the amino acids cystathionine, homocysteine, and cysteine by the action of at least two distinct enzymes: cystathionine-*γ*-lyase and cystathionine-*β*-synthase. In the past few years, H_2_S has emerged as a novel and increasingly important biological mediator. Imbalances in H_2_S have also been shown to be associated with various disease conditions. However, defining the precise pathophysiology of H_2_S is proving to be a complex challenge. Recent research in our laboratory has shown H_2_S as a novel mediator of inflammation and work in several groups worldwide is currently focused on determining the role of H_2_S in inflammation. H_2_S has been implicated in different inflammatory conditions, such as acute pancreatitis, sepsis, joint inflammation, and chronic obstructive pulmonary disease (COPD). Active research on the role of H_2_S in inflammation will unravel the pathophysiology of its actions in inflammatory conditions and may help develop novel therapeutic approaches for several, as yet incurable, disease conditions.

## 1. Introduction

Hydrogen sulfide (H_2_S) is a colorless, flammable, water-soluble gas characterized by a peculiar smell of rotten eggs. The toxic effects of H_2_S have been known for more than 300 years, and it has long been believed to be just an environmental pollutant [1]. In recent years, involvement of H_2_S is increasingly being recognized in several physiologic processes and disease states. H_2_S is produced endogenously in mammals including humans. In particular, cystathionine-*β*-synthase (CBS) in the central nervous system and cystathionine-*γ*-lyase (CSE) in the cardiovascular system are the key enzymes mostly responsible for the endogenous generation of H_2_S. Both enzymes use L-cysteine as the main substrate and pyridoxal-5′-phosphate as a cofactor [2]. Other enzymes responsible for the synthesis of H_2_S include (1) cysteine aminotransferase (CAT, EC 2.6.1.3), which catalyzes the reaction of l-cysteine with a ketoacid (e.g., *α*-ketoglutarate) to form 3-mercaptopyruvate and an amino acid such as l-glutamate-3-mercaptopyruvate may then be desulfurated by 3-mercaptopyruvate sulfurtransferase (MPST, EC 2.8.1.2) to form H_2_S and pyruvate; (2) cysteine lyase (EC 4.4.1.10), which converts l-cysteine and sulfite to l-cysteate and H_2_S [3]. As the end product of CBS- and CSE-catalyzed cysteine metabolism, H_2_S exerts a negative feedback effect on the activity of these enzymes [1, 4]. H_2_S has been shown to act as a modulator of N-methyl-D-aspartate receptor currents and hippocampal long-term potentiation. It also opens adenosine triphosphate-dependent K^+^ channel and interacts with other vasoactive gaseous mediators to relax smooth muscle [4–7]. There is evidence for the formation of a novel nitrosothiol from the interaction of H_2_S with nitric oxide (NO), a well-known gaseous mediator [8]. This nitrosothiol appears to play an important role in the action of H_2_S [9].

H_2_S *in vivo* is metabolized rapidly by a multitude of different chemical and enzymatic processes. Of these, the mitochondrial oxidation mechanism represents the most important route of H_2_S catabolism. It involves several enzymatic steps catalyzed by quinine oxidoreductase, S-dioxygenase, and S-transferase and, overall, leads to the formation of thiosulfate. Subsequently, thiosulfate is converted to sulfite by rhodanase and finally the major and stable product, sulfate, by sulfite oxidase [10, 11]. In contrast, cytosolic methylation of H_2_S by thiol-S-methytransferase to yield methanethiol and dimethylsulfide represents another less important mechanism of H_2_S degradation, and therefore accounts for a smaller amount of H_2_S [12]. Additionally, H_2_S can be scavenged by methaemoglobin to form sulfhaemoglobin or consumed by metallo- or disulfide-containing molecules such as oxidized glutathione [13, 14]. It can also be oxidized by activated neutrophils to form sulfite [15]. Finally, H_2_S is an endogenous reducing agent which can be easily consumed by a variety of circulating oxidant species in the vasculature such as peroxynitrite, hypochlorite, superoxide, or hydrogen peroxide [16–19]. It is excreted mainly by the kidney as free or conjugated sulfate [13].

In this paper, recent evidence that points to a key role of endogenously produced H_2_S as a novel mediator of inflammation is discussed.

## 2. Inflammation

Inflammation is a highly orchestrated, tissue-based response to traumatic, infectious, postischemic, toxic, or autoimmune injury. What Celsus defined around AD40 as “rubor, calor, dolor, tumor” (redness, heat, pain, and swelling) is today an intellectually engaging problem in systems biology, as well as a multibillion dollar market for the pharmaceutical industry. When primary pathogenic events are unknown, control of inflammation is sometimes the next best option. Initiated by responsive leukocytes and lymphocytes, a key component of the process is the trafficking of inflammatory cells to the sites of injury or infection. The cytokine/receptor interactions on the surface of these cells culminate in the expression of new gene products that efficiently kill or injure the invading organisms. However, uncontrolled production of inflammatory products is injurious to host cells and even leads to neoplastic transformation. Therefore, endogenous mechanisms have evolved to limit the production of inflammatory molecules and permit the resolution of the inflammatory response. In-depth studies of these mechanisms are important because defects in the pathway may contribute to the progression of chronic inflammatory disorders, and the pathway itself may present targets for novel anti-inflammatory therapeutic strategies [20–24]. 

Over the years, various studies have indicated a role of H_2_S in the inflammatory process. Reactive oxygen species from activated neutrophils could oxidize H_2_S to form sulfite, which then acts to upregulate leukocyte adhesion and neutrophil functions, through activation of Mac-1 *β*2-integrin (CD11b/CD18) and protein kinase C (PKC)/Ca^2+^-calmodulin pathway, respectively [15, 25–27]. Administration of nicotinamide adenine dinucleotide phosphate (NADPH) oxidase inhibitor suppressed whilst exogenous application of sodium hydrosulfide (NaHS), an H_2_S donor, enhanced these responses [26]. In addition, H_2_S provoked the short-term survival of granulocytes via inhibition of caspase-3 cleavage and p38 mitogen-activated protein kinase (MAPK) activation and therefore contributed to the bactericidal activity of neutrophils [28].

Recent work in our laboratory and others has shown a key role of H_2_S as a mediator of inflammation in different clinical conditions.

## 3. Acute Pancreatitis and Associated Lung Injury

Acute pancreatitis is a common clinical condition whose incidence has been increasing over recent years [29–33]. In United States alone, >300,000 patients are hospitalized annually with acute pancreatitis, leading to 3,200 deaths. Acute pancreatitis is a contributing factor in an additional 4,000 deaths annually. It also inflicts a heavy economic burden; the direct cost in the United States alone is >$2 billion annually [29–33]. In a majority of patients the condition is mild but 25% of patients suffer a severe attack and between 30 to 50% of these will die [29–33]. Most cases are secondary to biliary disease or excess alcohol consumption. The events that regulate the severity of acute pancreatitis are, for the most part, unknown. The exact mechanisms by which diverse etiological factors induce an attack are still unclear, but once the disease process is initiated common inflammatory and repair pathways are invoked. There is a local inflammatory reaction at the site of injury, which if marked leads to systemic inflammatory response syndrome (SIRS), and it is this systemic response that is believed to be ultimately responsible for the majority of the morbidity and mortality [29–33].

Since both CBS and CSE, two major H_2_S forming enzymes, are highly expressed in pancreatic acinar cells, it is of interest to understand the potential role of H_2_S in acute pancreatitis. Our group was the first to show the role of endogenously produced H_2_S as a mediator of inflammation [34]. We showed that mRNA for CSE is expressed in mouse pancreas and that pancreas homogenates convert L-cysteine to H_2_S *ex vivo*. Also, plasma levels of H_2_S are increased in mice upon induction of acute pancreatitis. The conversion of L-cysteine to H_2_S in pancreas homogenates was significantly reduced in mice pretreated with DL-propargylglycine (PAG) [34]. In addition, we showed that treatment of animals with PAG (either prophylactic or therapeutic) reduces the severity of pancreatitis as evidenced by a significant attenuation of hyperamylasemia, acinar cell injury/necrosis, and pancreatic myeloperoxidase (MPO) activity and by histological evidence of diminished pancreatic injury. Severe, but not mild, acute pancreatitis is associated also with lung injury, which is characterized by sequestration of neutrophils within the lung (i.e., increased lung MPO activity) and histological evidence of lung injury. In this study [34], we demonstrated that prophylactic/therapeutic administration of PAG additionally protected mice against acute pancreatitis associated lung injury as evidenced by a significant attenuation of lung MPO activity and by histological evidence of diminished lung injury (alveolar thickening and leukocyte infiltration) [34]. These effects of CSE blockade suggested an important proinflammatory role of H_2_S in regulating the severity of pancreatitis and associated lung injury and raise the possibility that H_2_S may exert similar activity in other forms of inflammation [34]. More recently, we have shown an important role of CBS in the pathogenesis of acute pancreatitis and associated lung injury [35]. In this study [35] we showed the presence of endogenous as well as caerulein stimulated production of H_2_S and ammonia (NH_3_) in the pancreas and lung. Caerulein increased plasma and tissue H_2_S and NH_3_ compared to saline control. Prophylactic or therapeutic administration of aminooxyacetate (AOA), a reversible inhibitor of CBS, directly inhibits CBS in the pancreas thereby reducing H_2_S and NH_3_ production, and protects against acute pancreatitis, further confirming the role of both the enzymes in inflammation in acute pancreatitis [35].

In isolated pancreatic acinar cells, the level of H_2_S and CSE mRNA were also significantly elevated in pancreatic acinar cells stimulated by caerulein [36]. Inhibition of H_2_S formation by PAG reduced mRNA expression and production of monocyte chemoatractant protein (MCP)-1, macrophage inflammatory protein- (MIP-) 1*α*, and MIP-2 in caerulein-stimulated mouse pancreatic acinar cells [36, 37]. Caerulein-induced acute pancreatitis was associated with a significant increase in MCP-1, MIP-1*α* and MIP-2 mRNA in both the pancreas and lungs, suggesting that they are important early mediators in both local as well as distant inflammatory response [37]. Blockade of H_2_S biosynthesis with PAG ameliorates the development of inflammatory process in caerulein-induced acute pancreatitis, acting through downregulation of chemokine expression [36, 37].

Furthermore, recent work in our laboratory has shown that H_2_S induces intercellular adhesion molecule-(ICAM-) 1 expression and neutrophil adhesion to caerulein-treated pancreatic acinar cells through nuclear factor- (NF-) *κ*B and Src-family kinases (SFK) pathway [38]. Results in this study [38] showed that H_2_S enhances ICAM-1 expression in cearulein hyperstimulated pancreatic acini and that this action involves SFK family phosphorylation. H_2_S activates SFKs in acinar cells and inhibition of SFKs impairs H_2_S-induced ICAM-1 expression secondary to the inhibition of NF-*κ*B activation. The effect of SFK inhibition on NF-*κ*B activation occurs together with I*κ*B*α* degradation. The results further demonstrate that neutrophil attachment onto H_2_S-treated acinar cells is increased and that inhibition of SFK function inhibits H_2_S-induced neutrophil attachment onto acinar cells. Taken together, these data indicate that H_2_S engages SFKs in order to signal ICAM-1 expression by a mechanism involving induction of NF-*κ*B. Results in this study [38] are consistent with a model wherein H_2_S activation of I*κ*B*α* and SFKs acts in concert to promote NF-*κ*B activity and ICAM-1 expression. Taken together, we proposed the mechanism by which H_2_S regulates ICAM-1 production in pancreatic acinar cells. Given the crucial role of ICAM-1 in facilitating the recruitment of neutrophil to acinar cells, targeting SFKs may be a useful strategy for suppressing the H_2_S-activated inflammatory response. These results highlight that SFKs may be an attractive therapeutic target for the treatment of acute pancreatitis. Also, results indicate a key role of the phosphatidylinositol 3-kinase-protein kinase B pathway in relation to the action of H_2_S on caerulein-induced cytokine production in isolated mouse pancreatic acinar cells [39].

Intraperitoneal administration of NaHS, an H_2_S donor, to mice caused a significant increase in circulating levels of substance P in a dose-dependent manner [40]. H_2_S, by itself, could also cause lung inflammation, as evidenced by a significant increase in lung MPO activity and histological evidence of lung injury. The maximum effect of H_2_S on substance P levels and on lung inflammation was observed 1 h after NaHS administration. At this time, a significant increase in lung levels of tumor necrosis factor (TNF)-*α* and interleukin (IL)-1*β* was also observed. In preprotachykinin (PPT)-A^−/−^ mice genetically deficient in substance P, H_2_S did not cause any lung inflammation. Furthermore, pretreatment of mice with CP-96345, an antagonist of the neurokinin-1 receptor (NK-1R) protected mice against lung inflammation caused by H_2_S. However, treatment with antagonists of NK-2, NK-3, and calcitonin gene related peptide (CGRP) receptors did not have any effect on H_2_S-induced lung inflammation. Depleting neuropeptide from sensory neurons by capsaicin significantly reduced the lung inflammation caused by H_2_S. In addition, pretreatment of mice with capsazepine, an antagonist of the transient receptor potential vanilloid-1 (TRPV-1), protected mice against H_2_S-induced lung inflammation. These results demonstrated a key role of substance P and neurogenic inflammation in H_2_S-induced lung injury in mice [40].

Substance P has been shown to play a key role in inflammation in acute pancreatitis [41–51]. Substance P treatment of isolated pancreatic acini results in an activation of chemokine synthesis, in turn resulting in an activation of inflammatory response [52–56]. Furthermore, substance P induces chemokine synthesis from macrophages and neutrophils, both of which are key players in inflammation [57–60].

In acute pancreatitis, PAG, given prophylactically as well as therapeutically, significantly reduced substance P concentrations in plasma, pancreas, and lung [61]. Moreover, prophylactic as well as therapeutic administration of PAG significantly reduced PPT-A mRNA expression and NK-1R mRNA expression in both pancreas and lung when compared with caerulein-induced acute pancreatitis. This reduction in PPT-A mRNA expression and NK-1R mRNA expression was associated with a protection against acute pancreatitis and associated lung injury. The increase in substance P production and NK-1R gene expression in the pancreas and lungs leads to increased inflammation and tissue injury in the pancreas and lung as evidenced by hyperamylasemia, myeloperoxidase (MPO) activities, and histological examination of the tissue injury. In this study [61] although pulmonary H_2_S synthesizing activity was not increased in caerulein group, increased plasma H_2_S due to increased pancreatic H_2_S synthesizing activity may upregulate PPT-A and NK-1R mRNA expression in lung and thereby caused increased production of SP in lung. Although inhibition of CSE enzyme activity had no impact on pulmonary H_2_S synthesizing activity, it caused significant reduction in pulmonary SP in acute pancreatitis. These results suggested that the proinflammatory effects of H_2_S may be mediated by SP-NK-1R pathway in acute pancreatitis [61]. In isolated pancreatic acini [36], inhibition of endogenous production of H_2_S by PAG significantly suppressed caerulein-induced increase in substance P concentration, PPT-A expression, and NK-1R expression. To determine whether H_2_S itself provoked inflammation in acinar cells, the cells were treated with NaHS, that resulted in a significant increase in substance P concentration and expression of PPT-A and NK-1R. Furthermore, we have shown that PPTA deficiency and blockage of H_2_S synthesis may play an important role that can regulate the toll-like receptor 4 (TLR4) pathway and subsequent innate immune response in acute pancreatitis, implying an interaction between SP/H_2_S occurs via TLR4 and NF-kB pathway. PPT-A gene deletion regulates H_2_S-induced TLR 4 signaling pathway in caerulein-treated pancreatic acinar cells, suggesting that in acute pancreatitis, H_2_S may upregulate the TLR4 pathway and NF-*κ*B via substance P [62]. The findings with these two proinflammatory mediators H_2_S and SP in TLR4 pathway has a crucial role in pro-inflammatory responses is not only important for understanding the basic mechanisms of H_2_S/TLR4-mediated gene activation but may also have implications for the development of anti-inflammatory drugs. Elucidation of the role of H_2_S/TLR4 in adaptive immunity might also open new opportunities for future treatment modalities in inflammation [62].

## 4. Sepsis

Sepsis is defined as the presence of bacteria or their toxins in blood or tissue and the systemic response that follows. Sepsis leading to at least one organ failure characterizes severe sepsis and septic shock is defined by severe sepsis accompanied by hypotension unresponsive to fluid resuscitation. Severe sepsis and septic shock are one of the leading causes of mortality among intensive care units and postoperative care patients [63–66]. The incidence of sepsis in North America has been reported to be 3.0 per 1,000 population, which transforms into an annual number of 750,000 cases, with 210,000 of them being fatal and a large socioeconomic burden [63–66]. The incidence of mortality due to sepsis is increasing and the most likely causes are the increased incidence of resistant pathogens and the advances of medical and surgical procedures that save the lives of many patients but leave them immunocompromized and in a state highly susceptible to death from severe sepsis and septic shock [63–66]. Sepsis and the events that follow are stages in a progressive condition—a systemic response to infection brought about by various inflammatory mediators, such as cytokines and chemokines. A relationship between cytokine cascades and sepsis has long been established but new evidence suggests that adhesion molecules also play a key role.

Using a clinically relevant model of cecal-ligation-and-puncture- (CLP-) induced sepsis, we have shown that H_2_S acts as a mediator of inflammation in this condition [67]. CLP-induced sepsis significantly increased the plasma H_2_S level and the liver H_2_S synthesis 8 h after CLP compared with sham operation. Induction of sepsis also resulted in a significant upregulation of CSE mRNA in liver. On the other hand, prophylactic as well as therapeutic administration of PAG significantly reduced sepsis-associated systemic inflammation, as evidenced by decreased MPO activity and histological changes in lung and liver, and attenuated the mortality in CLP-induced sepsis. Injection of NaHS, an H_2_S donor, significantly aggravated sepsis-associated systemic inflammation. Therefore, the effect of inhibition of H_2_S formation and administration of NaHS suggests that H_2_S plays a proinflammatory role in regulating the severity of sepsis and associated organ injury. Subsequent studies have shown the mechanism by which H_2_S contributes to inflammation in sepsis. For example, in one study [68] both prophylactic and therapeutic administration of PAG significantly reduced the mRNA and protein levels of IL-1*β*, IL-6, TNF-*α*, MCP-1, and MIP-2 in lung and liver, coupled with decreased nuclear translocation and activation of NF-*κ*B in lung and liver. Inhibition of H_2_S formation also significantly reduced lung permeability and plasma alanine aminotransferase activity. In contrast, injection of NaHS significantly aggravated sepsis-associated systemic inflammation and increased NF-*κ*B activation. In addition, H_2_S-induced lung inflammation was blocked by the NF-*κ*B inhibitor BAY 11-7082. Therefore, H_2_S upregulates the production of proinflammatory mediators and exacerbates the systemic inflammation in sepsis through a mechanism involving NF-*κ*B activation. In another study [69], using intravital microscopy, we found that in sepsis, prophylactic and therapeutic administration of PAG reduced leukocyte rolling and adherence significantly in mesenteric venules coupled with decreased mRNA and protein levels of adhesion molecules (ICAM-1, P-selectin, and E-selectin) in lung and liver. In contrast, injection of NaHS upregulated leukocyte rolling and attachment significantly, as well as tissue levels of adhesion molecules in sepsis. Conversely, in normal mice given NaHS to induce lung inflammation, NaHS treatment enhanced the level of adhesion molecules and neutrophil infiltration in lung. These alterations were reversed by pretreatment with BAY 11-7082. Moreover, expression of the chemokine receptor CXCR2 in neutrophils obtained from H_2_S-treated mice was upregulated significantly, leading to an elevation in MIP-2-directed migration of neutrophils. Therefore, H_2_S acts as an important endogenous regulator of leukocyte activation and trafficking during an inflammatory response. In a more recent study [70], we have shown that H_2_S regulates inflammatory response by activating the extracellular signal related kinase (ERK) pathway in polymicrobial sepsis. In this study, CLP-induced sepsis resulted in a time-dependent increase in the synthesis of endogenous H_2_S. Maximum phosphorylation of ERK1/2 and degradation of I*κ*B*α* in lung and liver were observed 4 h after CLP. Inhibition of H_2_S formation by PAG significantly reduced the phosphorylation of ERK1/2 in lung and liver 4 h after CLP, coupled with decreased degradation of I*κ*B*α* and activation of NF-*κ*B. In contrast, injection of NaHS significantly enhanced the activation of ERK1/2 in lung and liver, therefore leading to a further rise in tissue NF-*κ*B activity. As a result, pretreatment with PAG significantly reduced the production of cytokines and chemokines in sepsis, whereas exogenous H_2_S greatly increased it. In addition, pretreatment with PD98059, an inhibitor of MEK-1, significantly prevented NaHS from aggravating systemic inflammation in sepsis. This study, therefore, showed that H_2_S may regulate systemic inflammatory response in sepsis via the ERK pathway. In human monocyte U937 cells, H_2_S stimulates cell activation with the generation of proinflammatory cytokines, and this response is, at least partially, through the ERK-NF-*κ*B signaling pathway [71]. Similar to CLP-induced sepsis, a pro-inflammatory action of H_2_S was also observed in lipopolysaccharide (LPS)-induced endotoxemia [72, 73]. 

As in acute pancreatitis, substance P has been shown to play a key role in inflammation in sepsis [74–79]. An interaction between H_2_S and substance P was shown in a study in which PAG pretreatment or posttreatment significantly decreased the PPT-A gene expression and the production of substance P in lung, whereas administration of NaHS resulted in a further rise in the pulmonary level of substance P in sepsis. PPT-A gene deletion and pretreatment with the NK-1R antagonist L703606 prevented H_2_S from aggravating lung inflammation. In addition, septic mice genetically deficient in PPT-A gene or pretreated with L703606 did not exhibit further increase in lung permeability after injection of NaHS [80]. These findings showed that in sepsis, H_2_S upregulates the generation of substance P that contributes to lung inflammation and lung injury mainly via activation of the NK-1R. In a more recent study, we have shown that H_2_S induces systemic inflammation and multiple organ damage characteristic of sepsis via transient-receptor-potential-vanilloid-type-1-(TRPV1-) mediated neurogenic inflammation [81]. When given subcutaneously 30 minutes before CLP, the TRPV1 capsazepine treatment significantly attenuated systemic inflammation and multiple organ damage caused by sepsis, as characterized by lowered lung and liver MPO activities and histological evidence of diminished pulmonary and hepatic injury. Moreover, capsazepine delayed the onset of lethality and protected against sepsis-associated mortality. Administration of NaHS exacerbated but capsazepine reversed these deleterious effects. In the presence of PAG, capsazepine caused no significant changes to the PAG-mediated attenuation of systemic inflammation, multiple organ damage, and mortality in sepsis. More importantly, capsazepine has no effect on endogenous generation of H_2_S, suggesting that H_2_S is located upstream of TRPV1 activation, and may play a critical role in regulating the production and release of sensory neuropeptides in sepsis. Therefore, this study showed for the first time that H_2_S induces systemic inflammation and multiple organ damage characteristic of sepsis via TRPV1-mediated neurogenic inflammation [81]. Recent results [82] have identified the endogenous neural mediator that implicates in H_2_S-induced neurogenic inflammation and the molecular mechanisms by which H_2_S promotes TRPV1-mediated neurogenic inflammation in sepsis. In this study, capsazepine treatment resulted in a significant attenuation of circulating and pulmonary levels of substance P in septic mice. Capsazepine also inhibited NaHS-augmented substance P production but had no effect on PAG-mediated abrogation of substance P levels in both plasma and lung. Furthermore, capsazepine significantly reduced H_2_S-induced inflammatory cytokines, chemokines, and adhesion molecules expression, and protected against lung and liver dysfunction in sepsis. In the absence of H_2_S, capsazepine caused no significant changes to the PAG-mediated attenuation of sepsis-associated systemic inflammatory response and multiple organ dysfunction. Additionally, capsazepine greatly inhibited phosphorylation of ERK1/2 and I*κ*B*α*, concurrent with suppression of NF-*κ*B activation even in the presence of NaHS. In contrast, capsazepine had no effect on PAG-mediated abrogation of these levels in sepsis. Taken together, results in this study showed that H_2_S regulates TRPV1-mediated neurogenic inflammation in polymicrobial sepsis through enhancement of SP production and activation of the ERK-NF-*κ*B pathway [82]. Also, a recent study has shown that H_2_S upregulates cyclooxygenase-2 (COX-2) and prostaglandin E metabolite [PGEM] in sepsis-evoked acute lung injury via TRPV-1 channel activation [83]. Results in this study [83] demonstrated that induction of sepsis by CLP resulted in a concomitant and significant overproduction of COX-2 and PGEM in the lungs. Administration of NaHS further enhanced the biosynthesis of COX-2 and PGEM whereas PAG significantly decreased these levels. The use of two different yet complementary approaches in this work: exogenous administration of NaHS that serves as an H_2_S donor and inhibition of endogenous H_2_S synthesis by PAG which acts as an irreversible inhibitor of CSE, directly ascertained the involvement of H_2_S in augmenting the production of COX-2 and PGEM in sepsis. The use of capsazepine, a selective TRPV1 antagonist, determined that H_2_S upregulates COX-2 and PGEM in sepsis by a TRPV1 channel-dependent mechanism. Additionally, since blockade of TRPV1 by capsazepine attenuated H_2_S-augmented COX-2 and PGEM response but had no effect on PAG-mediated attenuation of both parameters in septic lungs, we ascertained from our data that sepsis has a significant sensory neurogenic component that is mediated by H_2_S in a TRPV1-dependent manner, and that H_2_S stimulation of TRPV1 occurs upstream of COX-2 and PGEM in sepsis [83]. Furthermore, COX-2 inhibition with parecoxib significantly attenuated pulmonary neutrophil infiltration, edema formation, production of inflammatory cytokines, chemokines, and adhesion molecules, as well as restored lung histoarchitecture in sepsis, thereby significantly prevented the development of sepsis-evoked acute lung injury. Moreover, the fact that exogenous administration of NaHS to septic mice showed exacerbated acute lung injury compared to mice subjected to CLP alone, and that these pathophysiologic consequences of acute lung injury were significantly abrogated upon treatment with parecoxib confirm the notion that sepsis-evoked acute lung injury was specifically attributable to the interplay between H_2_S and COX-2. Intrigued by the strong inhibitory effects of parecoxib on H_2_S-induced inflammation and injury in the lungs of septic mice, our results also suggest that selective blockade of COX-2 could constitute an important therapeutic target for the treatment of sepsis-evoked acute lung injury [83]. This report, therefore, showed that H_2_S augments the upregulation of COX-2 and PGEM, which orchestrates the neurogenic inflammatory response via activation of TRPV1 channel, and consequently contributes to lung inflammation and injury in a mouse model of sepsis-induced ALI. Additionally, inhibition of COX-2/PGE_2_ pathway in the septic lungs significantly ameliorates inflammation, injury and sepsis-associated mortality; thereby provide a potential therapeutic approach for the prevention of ALI in sepsis [83].

## 5. Burn Injuries

Burn injuries represent one of the most widespread and devastating forms of trauma and are ranked among the leading causes of morbidity and mortality worldwide with one of the highest burn death rates [84–86]. H_2_S has recently been shown to act as a critical mediator of severe burn injury-induced inflammation in mice [87]. The result in this study show that burn injury in mice subjected to 25% total body surface area full thickness burn augmented significant increase in plasma H_2_S levels, liver, and lung CSE mRNA expression, and liver H_2_S synthesizing activity. Furthermore, the enhanced H_2_S/CSE pathway correlates with heightened burn-associated systemic inflammation, as evidenced biochemically and histologically by increased MPO activity and worsened histological changes in the lung and liver. Importantly, the development of systemic inflammation and multiple organ damage were mitigated when endogenous H_2_S synthesis was blocked by prophylactic or therapeutic treatment of PAG, along with attenuation of plasma H_2_S levels, liver, and lung CSE mRNA expression, and liver CSE synthesizing activity. Administration of NaHS at the same time of burn injury resulted in a further rise in MPO activity and more severe tissue injury in the lung and liver. Collectively, these findings have shown for the first time the role of H_2_S in contributing to exaggerated inflammatory damage after burn injury [87]. The mechanism by which H_2_S contributes to inflammation remains to be investigated, although, in light of the role of substance P in inflammation in burn injuries [88–90], a potential contribution of substance P in H_2_S-induced inflammation in burns is highly likely.

## 6. Joint Inflammation

Joint inflammation, that clinically manifests as arthritis, is a major health problem worldwide [91–94]. The possible role of endogenous H_2_S in the development of joint inflammation has recently been investigated. In these studies, edema was induced by carrageenan in rodent hindpaws. An increase in H_2_S synthesis in inflamed hindpaws was seen, suggesting a localized overproduction of H_2_S during inflammation [95]. Pretreatment with PAG resulted in a dose dependent inhibition of hindpaw edema as well as hindpaw MPO activity. These findings suggest that H_2_S is an endogenous mediator of the development of hindpaw local inflammation. This and other studies, however, have been done in experimental animal models of disease or in cell lines and their clinical relevance remains unclear. Recent reports in the literature, albeit with conflicting results, point to a role of H_2_S in rheumatoid arthritis [96, 97]. One of these studies [96] confirmed the pro-inflammatory action of H_2_S in rheumatoid arthritis, whereas the other [97] suggested an anti-inflammatory effect.

## 7. Chronic Obstructive Pulmonary Disease (COPD)

In addition to its role in acute inflammatory diseases, a H_2_S appears to be an important mediator in chronic inflammatory disease as well. Chronic obstructive pulmonary disease (COPD) is a common clinical condition characterized by progressive airflow obstruction that is only partly reversible, inflammation in the airways, and systemic effects or comorbities. The main cause is smoking tobacco, but other factors have been identified [98]. Serum H_2_S levels were significantly increased in patients with stable COPD as compared to age matched control subjects or those with acute exacerbation of COPD (AECOPD) [99]. Serum H_2_S levels were negatively correlated with the severity of airway obstruction in patients with stable COPD whereas they were positively correlated with the lung function in all patients with COPD and healthy controls. Patients with AECOPD and increased pulmonary artery pressure (PASP) had lower levels of H_2_S than those with normal PASP, suggesting a negative relation between H_2_S and PASP in AECOPD patients. Interestingly, they also found that serum H_2_S levels were lower in smokers than non-smokers regardless of their health status (COPD or healthy controls). In addition, patients with AECOPD, whose serum H_2_S levels were decreased, had greater neutrophil proportion but lower lymphocyte proportion in sputum than patients with stable COPD, suggesting a potential role of H_2_S in regulating inflammatory response at different types or stages of COPD. This study [99] demonstrated that endogenous H_2_S may participate in the development of airway obstruction in COPD and that the level of endogenous H_2_S may be correlated with the progression and severity of COPD.

## 8. H_2_S-Releasing Non-Steroidal Anti-Inflammatory Drugs (NSAIDs) and Slow H_2_S-Releasing Drugs: An Anti-Inflammatory Action of H_2_S? 

S-diclofenac (ACS 15) is H_2_S-releasing diclofenac, which comprises a H_2_S-releasing dithiol-thione moiety attached by an ester linkage to diclofenac. We have investigated [100] the effect of treatment with the NSAID diclofenac and its H_2_S-releasing derivative ACS15 on caerulein-induced acute pancreatitis and the associated lung injury. Although these two drugs did not have any significant effect on the local pancreatic injury in acute pancreatitis, ACS15 afforded significant protection against acute pancreatitis-associated lung injury. The protective action of this drug was apparent when administered either prophylactically or as a therapeutic treatment. ACS15 significantly attenuated the rise in lung MPO activity in severe acute pancreatitis. In addition, histological evidence showed significant protection against acute pancreatitis-associated lung injury [100]. ACS 15 also more effectively inhibited hindpaw swelling and neutrophil infiltration after carrageenan injection as compared to its parent NSAID [101]. Furthermore, in a rat model of LPS-induced endotoxemia, ACS 15 exhibited enhanced anti-inflammatory effect as compared to the parent drug [102]. Although these results suggest the potential for the use of controlled release H_2_S donor compounds against inflammation (and some literature may mistakenly suggest this), the protective action of ACS 15 in LPS-induced endotoxemia was associated with an inhibition of endogenous H_2_S synthesis [102]. Therefore, protective actions of H_2_S releasing compounds may be caused by an inhibition of endogenous H_2_S formation, possibly by a negative feedback mechanism caused by very low local levels of H_2_S. These results, therefore, further reinforce the pro-inflammatory action of endogenous H_2_S. Another H_2_S-releasing drug is S-propargyl-cysteine (SPRC), a structural analog of S-allyl cysteine (SAC) with a common cysteine-containing structure [103]. SPRC has been shown to have a good safety profile and cardioprotective action and has been reported to show protective effects against myocardial infarctions in both adult rat hearts and neonatal cardiomyocytes through H_2_S pathway [98–102]. Another slow H_2_S releasing compound is GYY4137 (morpholin-4-ium-4-methoxyphenyl(morpholino)phosphinodithioate), which has been reported to have antihypertensive and anti-inflammatory action [104–106]. In a recent study, SPRC 10 mg/kg injected 3 h prior to induction of acute pancreatitis ameliorated the disease by reducing the inflammatory cell infiltration in pancreas and lung and by modulating pro- and anti-inflammatory cytokine profile in plasma. Results showed that pancreatic injury, as evidenced by plasma amylase, pancreatic MPO and histology, and pulmonary injury, as evidenced by lung MPO and histology were significantly ameliorated in the mice treated with SPRC 3 h prior to the induction of pancreatitis. Prolonged increase in MPO activity is reported to indicate continued neutrophil activation, with the liberation of cytokines and other biologically active substances like reactive oxygen species. We observed a significant reduction in the pro-inflammatory cytokines (IL-1*β* and IL-6) in pancreas and lung of mice treated with SPRC 3 h before the induction of AP compared to the mice pretreated with vehicle. Furthermore, pancreatic and pulmonary anti-inflammatory cytokine interleukin-10 (IL-10) levels were significantly increased in mice pretreated with SPRC compared to vehicle treated group. Thus SPRC provides a valuable lead for the treatment of acute pancreatitis [107]. It could be postulated that the beneficial effects of SPRC in AP could be by virtue of its slow release of endogenous H_2_S and a possible negative feedback mechanism on CSE. 

In some studies, endogenous production and exogenous administration of H_2_S has also been reported to be anti-inflammatory in many models of pathologies including asthma [108], synovitis [109], LPS induced inflammation [110], rhinitis [111], ischemia reperfusion [112], neuropathologies [113], and gastric pathologies [114, 115]. The mechanisms of hydrogen sulfide's anti-inflammatory properties are still to be elucidated and may be more indirect than the pro-inflammatory effects, and may occur primarily with administration of lower concentrations and/or slow releasing compounds. Other possible mechanisms include hypothermia and tissue protection [23].

## 9. The Way Forward

In recent years, substantial basic science research has led to a reasonably clear understanding of the role of H_2_S as an inflammatory mediator. Several gaps in knowledge, however, remain. Firstly, a lot of the data on H_2_S in inflammation has been generated using inhibitors of H_2_S synthesis, such as PAG. While undoubtedly very useful pharmacological tools in a new but rapidly expanding research field, it is unlikely that compounds such as PAG are entirely specific since they target the pyridoxal phosphate binding site of CSE and CBS and other PLP-dependent and -independent enzymes [116–121]. Therefore, there is need to develop inhibitors of H_2_S synthesis, which are more selective and have a better safety profile than the ones that are currently available. Approaches using CSE knockout mice [122] would enable further define the role of H_2_S in inflammation. Also, clinical studies examining the significance of H_2_S in clinical inflammatory conditions are scarce. Despite it being in its infancy stage, early results in this direction have been rather promising and allow us to consider the clinical potential of developing drugs that interfere with H_2_S production. Indeed, efforts are underway to determine the therapeutic potential of targeting H_2_S in treatment of inflammatory pathologies. Nevertheless, our understanding of the molecular mechanisms by which H_2_S contributes to inflammation is still fragmentary. Active research in this direction will lead to further insights into the development of new therapeutic intervention for inflammatory conditions, and most importantly, to facilitate transition of our knowledge from the laboratory to the clinic.

## Abbreviations

H_2_S: Hydrogen sulfide;
CBS: Cystathionine-*β*-synthase;
CSE: Cystathionine-*γ*-lyase;
CAT: Cysteine aminotransferase;
MPST: 3-Mercaptopyruvate sulfurtransferase;
NO: Nitric oxide;
PKC: Protein kinase C;
NADPH: Nicotinamide adenine dinucleotide phosphate;
NaHS: Sodium hydrosulfide;
MAPK: Mitogen-activated protein kinase;
SIRS: Systemic inflammatory response syndrome;
PAG: DL-propargylglycine;
MPO: Myeloperoxidase;
NH_3_: Ammonia;
AOA: Aminooxyacetate;
MCP: Monocyte chemoatractant protein;
MIP: Macrophage inflammatory protein;
ICAM: Intercellular adhesion molecule;
NF: Nuclear factor;
SFK: Src-family kinases;
TNF: Tumor necrosis factor;
IL: Interleukin;
PPT: Preprotachykinin;
NK-1R: Neurokinin-1 receptor;
CGRP: Calcitonin gene related peptide;
TRPV-1: Transient receptor potential vanilloid-1;
ERK: Extracellular signal related kinase;
TLR4: Toll-like receptor 4;
COX-2: Cyclooxygenase-2;
PGEM: Prostaglandin E metabolite;
COPD: Chronic obstructive pulmonary disease;
AECOPD: Acute exacerbation of COPD;
PASP: Pulmonary artery pressure;
NSAIDs: Non-steroidal anti-inflammatory drugs;
SAC: S-allyl cysteine
SPRC: S-propargyl-cysteine;
CLP: Cecal ligation and puncture.
